# Integrating value of research into NCI Clinical Trials Cooperative Group research review and prioritization: A pilot study

**DOI:** 10.1002/cam4.1657

**Published:** 2018-07-20

**Authors:** Josh J. Carlson, David D. Kim, Gregory F. Guzauskas, Caroline S. Bennette, David L. Veenstra, Anirban Basu, Nathaniel Hendrix, Dawn L. Hershman, Laurence Baker, Scott D. Ramsey

**Affiliations:** ^1^ University of Washington Seattle Washington; ^2^ Tufts Medical Center Boston Massachusetts; ^3^ Flatiron Health New York New York; ^4^ Colombia University New York New York; ^5^ University of Michigan Ann Arbor Michigan; ^6^ Fred Hutchinson Cancer Research Center Seattle, Washington

## Abstract

**Background:**

The Institute of Medicine has called for approaches to help maximize the return on investments (ROI) in cancer clinical trials. Value of Research (VOR) is a health economics technique that estimates ROI and can inform research prioritization. Our objective was to evaluate the impact of using VOR analyses on the clinical trial proposal review process within the SWOG cancer clinical trials consortium.

**Methods:**

We used a previously developed minimal modeling approach to calculate VOR estimates for 9 phase II/III SWOG proposals between February 2015 and December 2016. Estimates were presented to executive committee (EC) members (N = 12) who determine which studies are sent to the National Cancer Institute for funding consideration. EC members scored proposals from 1 (best) to 5 based on scientific merit and potential impact before and after receiving VOR estimates. EC members were surveyed to assess research priorities, proposal evaluation process satisfaction, and the VOR process.

**Results:**

Value of Research estimates ranged from −$2.1B to $16.46B per proposal. Following review of VOR results, the EC changed their score for eight of nine proposals. Proposal rankings were different in pre‐ vs postscores (*P* value: 0.03). Respondents had mixed views of the ultimate utility of VOR for their decisions with most supporting (42%) or neutral (41%) to the idea of adding VOR to the evaluation process.

**Conclusions:**

The findings from this pilot study indicate use of VOR analyses may be a useful adjunct to inform proposal reviews within NCI Cooperative Clinical Trials groups.

## 1 INTRODUCTION

The National Academy of Medicine (formerly the Institute of Medicine has called for approaches to help maximize the return on research investments in cancer clinical trials, stating that, “prioritization and selection of trial concepts is critical to ensure that limited public funds are used in ways that are likely to have the greatest impact on patient care.”^1^ Value of Research (VOR; also known as Value of Information, VOI) analysis is a health economics technique that estimates the clinical and economic returns for research investments.^2^, ^3^, ^4^, ^5^, ^6^, ^7^ Specifically, VOR estimates the value of reducing treatment decision uncertainty, by comparing the evidence that exists for a therapy today vs the aggregated evidence generated by collecting additional information (eg, through a clinical trial). This estimate of the potential reduction in evidence uncertainty can inform policymakers of the sufficiency of current evidence to adopt a new therapy, as well as the remaining risk of prematurely making a “wrong” decision. Cancer clinical trials groups, with an abundance of testable and potentially impactful research ideas, coupled with their reliance on constrained public budgets, are an ideal testing ground to evaluate the addition of VOR estimates to their research prioritization processes.

As an example, consider the information available from a small clinical trial for a given cancer drug, with a modest treatment effect and a wide confidence interval given the limited size of the study population. If clinicians were to make treatment decisions based on this small trial, the probability that they would be making the optimal decision might be fairly low. By conducting an analogous, larger trial, with smaller confidence intervals for each outcome, the impact of the drug on the outcome and *the uncertainty about the result* falls substantially, thus our chances of making a correct treatment decision is increased—regardless of whether the trial is “positive” or “negative.” VOR analysis captures the value of the additional research by estimating the likely future impacts on patient outcomes and healthcare resources using economic theory and decision modeling techniques. VOR is particularly useful in prioritizing research when it can be applied in decision‐relevant time frame, can be customized to individual decision‐making groups, and produces a metric that is comparable across analyses; all else being equal, the research proposals with the highest VOR should be prioritized over others.^7^, ^8^, ^9^, ^10^

In the context of VOR, the economic value of a clinical trial is a function of four key elements: (a) the current level of decision uncertainty (ie, the probability that we are making suboptimal decisions based on current knowledge), (b) the scale and scope of new information to be collected in the trial, (c) the consequences of making a suboptimal decision in terms of a patient's life expectancy, quality of life, or healthcare costs, and (d) the number of future patients likely to face the decision. VOR for a particular study will be high when there is substantial uncertainty about the decision, the clinical and/or economic consequences of making a suboptimal choice are significant, and/or the affected population is large.

Although the theory and basic methodology of VOR analysis have been described for several years, practical use of VOR in real‐world research decision settings has been limited. Accordingly, we engaged with SWOG, a large cancer clinical trials network, to develop a cooperative group, clinical trial‐oriented process for integrating VOR estimates into the research prioritization process. The objectives of our study were to evaluate the impact of VOR estimates on the decisions made by SWOG's executive review committee (EC) and evaluate their opinions about VOR and its usefulness for their decision‐making criteria.

## 2 METHODS

### 2.1 Setting

This work was conducted as part of a Patient‐Centered Outcomes Research Institute (PCORI) funded project evaluating a structured approach to prioritizing cancer research using stakeholders and VOR within SWOG, one of four NCI‐sponsored clinical trials networks (NCTN). SWOG research studies are proposed and developed by members from committees (eg, lung, breast cancer) and, after approval by the organ‐based committee, study proposals are sent to SWOG's Executive Review Committee (EC) for an internal review. EC members assign a prioritization score to proposals after presentation by the study lead investigators. While the established evaluation and scoring process considers a large number of factors, EC members are asked to specifically address the following issues: (a) the scientific strength and feasibility of the proposal; (b) potential overlap with actively recruiting SWOG studies that might pose threats to accrual; (c) whether the study leverages other research currently being conducted in NCI‐supported cancer centers; and (d) potential future impact on cancer patient care and outcomes irrespective of the outcome of the study (ie, “positive” or “negative”). Highly scored proposals are sent to the Cancer Treatment Evaluation Program at the NCI. Lower score proposals are returned to the investigator for revisions, or are rejected for further development. Our study focused on phase II and III randomized studies from the breast, genitourinary (GU), gastrointestinal (GI), and cancer care delivery (CCD) committees.

### 2.2 Stakeholder training in VOR

Our process included training in VOR theory and methods. We engaged SWOG members from the EC and the included disease committees (approximately 200 total participants) in an iterative and multifaceted manner using in‐person meetings, web‐enabled teleconferences, and web‐based educational materials to provide training in VOR and actively solicit their preferences and feedback (see VOR educational materials in the Supplemental materials). Our goals were to create shared understanding of VOR methods and develop a transparent and SWOG‐specific process for generating and presenting VOR estimates as part of SWOG's proposal evaluation process.^11^

### 2.3 Generating and presenting VOR estimates for study concepts

**Figure 1:**
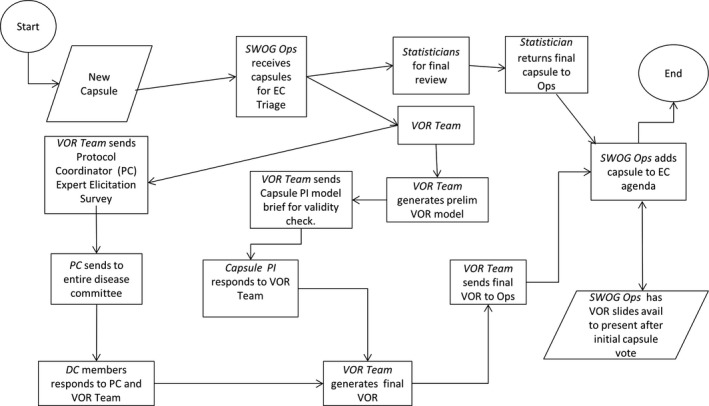
A Graphic Representation of the Process we used to Integrate VOR Analyses into SWOG's Proposal Evaluation Procedures

Our approach involved integration of VOR without undue burden on SWOG processes and timelines (Figure 1). After receipt of a proposal, we used a previously developed minimal modeling approach to calculate per‐patient and population‐level (based on US cancer incidence) VOR estimates.^8^ We estimated the level of uncertainty regarding the proposed trial using either expert elicitation or historical data. These approaches are described in more detail in a supplementary methods section (Appendix S1). We also performed a validation step in which we contacted the proposal's principal investigator to review the model inputs. We then developed a final model, generated, and presented full VOR results with details on the model structure, inputs, and VOR estimates.

The VOR estimates were provided in both disaggregated (ie, the expected clinical and economic impacts separately) and aggregated forms based on feedback during the development phase of the project. The aggregated forms included the development and use of clinical VOR (ie, VOR based solely on the expected clinical benefit), and comprehensive VOR (ie, VOR estimates based on expected clinical and economic impacts). The clinical and comprehensive VOR estimates were specific to the clinical study evaluated and used the expected value of sample information approach.^12^

### 2.4 Study sample

The prospective VOR evaluation phase was conducted from February 2015 to December 2016. A total of 10 studies met our initial criteria of randomized phase II or III studies from an included disease committee. One study was not reviewed by the EC due to external logistical factors for the study; thus nine studies were evaluated, presented, and scored (Table 1). EC committee members were provided access to training materials about VOR and our processes prior to (or during in the case of new members) the prospective evaluation phase.

**Table 1:** Value of research results and EC scores

Study #	Score PRE	Score POST	Study phase	Disease	Sample size	New patients per year	Patient VOR	Population VOR (billions)
1	2.50	3.40	II	Gastric	219	4000	−$70 840	−$1.1
2	2.75	2.88	II	Breast	276	4000	$157 673	$2.4
3	2.50	2.83	II	Pancreatic	132	10 000	$13 033	$0.73
4	2.88	3.38	II	Breast	60	1000	−$6502	−$0.033
5	2.50	2.56	III	Breast	1000	1200	−$28 497	−$0.067
6	2.75	2.50	III	Bladder	616	25 900	$28 422	$2.4
7	2.22	2.50	II	Colorectal	86	3000	−$66 106	−$1.1
8	3.00	4.00	II	Colorectal	102	26 540	−$14 654	−$2.1
9	4.00	4.00	III	Cancer of unknown primary	600	15 400	$292 360	$16.4

### 2.5 Value of research (VOR) calculations

We estimated the expected VOR using Bayesian decision theoretic methods. Our methods have been described previously,^8^ but briefly, we (a) created a minimal decision model using a simple Markov model framework; (b) populated the model with data from the clinical trial proposal and external data sources; (c) characterized the level of current uncertainty including the prior distribution of the treatment effect; (d) simulated the range of expected trial results using the current level of uncertainty and the trial's planned sample size and length of follow‐up; (e) compared the expected quality‐adjusted life years (QALYs) gained and healthcare costs of decisions made with the additional evidence from the proposed trial to those made with only existing evidence; and (f) estimated the size of the relevant patient population expected to face the treatment decision using the Surveillance, Epidemiology, and End Results (SEER) database and published literature. Analyses were performed in Microsoft Excel© or R.^13^ We assumed a time horizon of 10‐years for the information being generated in each trial to be decision‐relevant.^14^, ^15^ We also used a 3% discount rate for future costs and benefits, and accounted for the delay in the availability of information by including the accrual rates and specified follow‐up time in the trial proposals.^16^

### 2.6 Minimal modeling framework

We developed and used a Markov model framework using the trial proposal's primary endpoint. This framework consisted of up to three health states: (a) alive, preprimary endpoint, (b) alive, postprimary endpoint, and (c) death, and was informed by work by Meltzer and Basu and has been described previously.^8^, ^10^ This modeling framework is considered sufficient and appropriate for the research prioritization context given the need for timely model development and the availability well‐developed study capsules that include evidence and expert opinion to empirically characterize the relationship between the trial's primary endpoint and a comprehensive measure of health outcomes.^8^, ^10^ We estimated the probability of transitioning from preprimary endpoint to postprimary endpoint for the control arm from the survival parameters included in the trial proposal and assuming a constant failure rate (ie, an exponential distribution). This is in line with the assumptions used in the trial proposal's sample size calculations.

### 2.7 Executive review committee evaluation regarding opinions of VOR for decision making

Executive committee members scored proposals before and after receiving the full set of VOR estimates including the expected incremental QALYs and costs, the clinical VOR, and the comprehensive VOR, during SWOG's regularly scheduled proposal review meetings. Scores ranged from 1 (best) to 5.

To evaluate EC member's opinions about their experience with VOR we surveyed members at baseline and again at study end (Appendix S2: EC surveys). The surveys were informed by targeted telephone interviews with SWOG staff and EC members coupled with previous work evaluating stakeholder opinions about VOR.^9^, ^17^ The baseline survey included questions about research priorities during proposal evaluation and satisfaction with the current proposal evaluation process. The end‐of‐study survey also included additional questions about the VOR process. Survey participants were contacted via email and provided a link to the web‐based survey.

### 2.8 Data analysis

The primary outcomes were the EC proposal score and proposal ranking before and after viewing the VOR results. In secondary analysis, we evaluated the association between the per patient and population‐level VOR estimates and the change in scores. For both these analyses, we used the Wilcoxon signed‐rank test, which is a nonparametric statistical hypothesis test used for comparing repeated measurements to determine whether the population mean ranks differ.

We analyzed the survey results using descriptive statistics. For the subset of questions and respondents for whom we had baseline and postsurvey results, we evaluated the change in respondent answers about the importance of several decision‐making factors using the Wilcoxon signed‐rank test. All statistical tests were two‐sided using an alpha level of 0.05.

## 3 RESULTS

**Figure 2:**
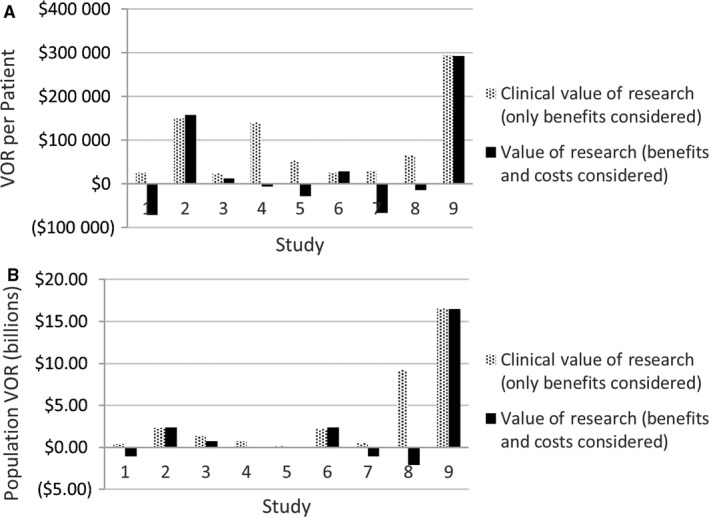
The VOR Estimates for each Proposal at the per Patient and Population Level and Using the Comprehensive and Clinical VOR Metrics

**Figure 3:**
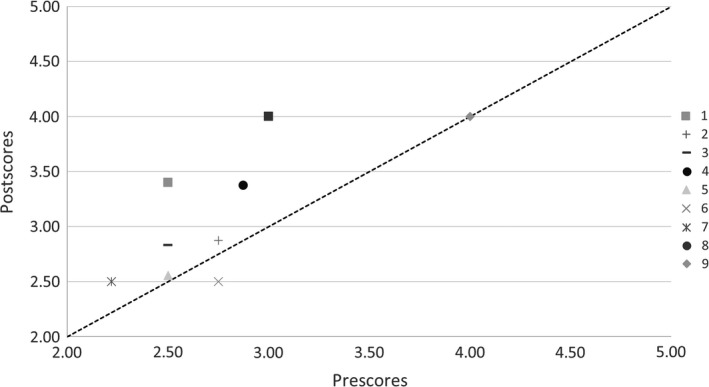
The Executive Committee Proposal Scores Pre‐ and Postreceiving the VOR Estimates. The Capsule Rankings were Different in the Pre vs Postscores, Implying that a Different set of Capsules may have been Prioritized Under a Fixed Budget

Among the nine studies evaluated, six were phase II and three were phase III, target sample sizes ranged from 60 to 1000, and the disease areas involved were breast (3), colorectal (2), gastric (1), pancreatic (1), bladder (1), and cancer of unknown primary (1). The VOR results (Table 1, Figures 2 and 3) show that the population clinical VOR estimates ranged from $0.13B to $16.53B and the comprehensive VOR estimates ranged from −$2.1B to $16.46B. The EC proposal scores changed for eight of nine proposals following presentation of VOR results. Proposal rankings were significantly different in the pre‐ vs postscores (*P* value: 0.03). The scores for six of the nine proposals changed in the direction of the comprehensive VOR estimate (ie, the score went down indicating a higher rank, when the VOR was positive indicating a positive return on investment), one did not change and two moved in the opposite direction. However, there was no significant association between comprehensive VOR estimates and the magnitude of the change in proposal scores (*P* > 0.05). We did not find an association between the direction or the magnitude of the VOR estimates and the direction or magnitude of the proposal score change (all *P* > 0.1).

### 3.1 EC survey results

At baseline 11 of 16 EC members consented to and completed the baseline survey. At study end, there were 15 EC members (four were no longer on the EC and three added) 12 of which completed the follow‐up survey. Two did not consent and one was excluded due to her role as a study co‐investigator. Nine respondents completed both baseline and end‐of‐study surveys. Pooled respondent characteristics are provided in Table 2.

**Table 2:** Executive review committee characteristics

Professional experience	
Mean years (SD) in SWOG	17.9 (4.9)
Mean years (SD) on EC	4.2 (2.8)
Professional training (%)	
MD	64%
PhD	27%
Other	9%
Specialty (%)	
Breast cancer	35%
Genitourinary cancer	17%
Hematologic malignancies	13%
Radiation oncology	9%
Other	26%

There was a general trend toward decreased average ratings of importance for most of the listed factors; ratings for economic value increased (Table 3). The results of the poststudy survey are provided in Table 4 and Table 5. Sixty‐seven percent of respondents rated their knowledge about VOR as moderate or high at study end vs 0% prior to this study. Seventy‐five percent felt confident interpreting VOR data.

**Table 3:** Results of the Executive Review Committee survey pre/postanalysis

Pre/postanalysis	Baseline (n = 9) Mean	End of study (n = 9) Mean	Incremental change	*P* value^a^
Feasibility	6.56	5.78	−0.78	0.056
Clinical importance	6.33	5.89	−0.44	0.164
Scientific contribution	6.00	6.00	0.00	1
Relative resource use	4.89	5.00	0.11	0.95
Economic value	3.78	4.78	1.00	0.0168
Disease burden	4.44	4.22	−0.22	0.157
Current uncertainty	4.89	4.56	−0.33	0.472
Applicability to clinical practice	6.00	6.11	0.11	0.655
Timeliness	5.67	5.78	0.11	0.706
Suitability for SWOG	6.11	5.78	−0.33	0.083
Role of NCI	4.56	4.56	0.00	1
VOR	N/A	5.00	N/A	N/A

a Paired *T* test

**Table 4:** Results of the Executive Review Committee end‐of‐study survey on the VOR experience

Experience of the VOR process (n = 12)			
	Moderate/High, %		Low/No, %
Prior knowledge of VOR	0		100
Postknowledge of VOR	67		33
Confidence in interpreting VOR	75		25

	(Somewhat) agree, %	Neither agree nor disagree, %	(Somewhat) disagree, %
The team addressed my input before incorporating VOR	27	46	27
Training in VOR was sufficient	67	25	8
VOR material provided to EC was easy to understand	67	16	17
VOR material provided to EC was appropriate in length	91	9	0
The VOR proposal evaluation aided my decision making	50	42	8
The VOR materials helped the evaluation process	50	42	8
The VOR materials hindered the evaluation process	8	42	50
I support adding VOR to the proposal evaluation process	42	41	17

The results of the Executive Review Committee end‐of‐study survey. The questionnaire is provided in the Supplementary materials. The questions used a Likert scale.

**Table 5:** Results of the Executive Review Committee end‐of‐study survey: importance of factors related to decision making

Importance of factors in decision making (n = 12)							
	Not at all important, %	Very low, %	Slightly, %	Somewhat, %	Moderately, %	Very, %	Extremely, %
Feasibility	0	0	0	9	4	52	35
Clinical importance	0	0	0	9	17	43	31
Scientific contribution	0	0	0	9	18	55	18
Relative resource use	0	0	9	13	61	17	0
Economic value	0	9	22	17	35	17	0
Disease burden	0	0	18	39	30	13	0
Current uncertainty	0	0	9	22	56	4	9
Applicability to clinical practice	0	0	0	5	18	50	27
Timeliness	0	0	0	9	26	56	9
Suitability for SWOG	0	0	0	9	9	61	21
Role of NCI	0	9	13	13	52	13	0
VOR (post only)	0	0	9	9	55	27	0

Respondents had mixed views of the ultimate utility of VOR for their decisions but were mostly positive. Two‐thirds felt that the training was sufficient and that the VOR material was easy to understand. The majority (91%) felt that the VOR material provided to EC was appropriate in length. Most respondents either agreed (50%) or were neutral (42%) about whether the VOR proposal evaluation aided their decision making or helped the evaluation process. Forty two percent support adding VOR to the evaluation process with 41% neutral and 17% disagreeing.

## 4 DISCUSSION

As an experiment to aid decision making about clinical trial research prioritization, we developed and implemented a VOR evaluation process for SWOG, a large NCI‐sponsored cancer clinical trials cooperative research group. The process involved rapid development of VOR models based on the clinical trial research proposal, published literature, and expert elicitation followed by model validation. The content and format of the VOR results were developed with input from SWOG members. The results impacted scores for eight out of nine study proposals. While the implementation of the VOR process was feasible, EC members had mixed acceptance of the idea of integrating VOR into the proposal evaluation process: roughly half viewed it favorably and the remainder were neutral or opposed.

To our knowledge, this is the first study to develop processes for incorporating VOR methods and results into an established clinical trials prioritization review process in the United States. Encouragingly, we found that the clinicians who design and implement clinical trials readily grasped the concept of VOR and the implications of VOR for trial concept proposals during their weekly review and rating sessions. In addition, it was feasible to generate VOR results in the generally short window between the time when of trial proposal completes final statistical review after leaving the disease committee and the time it was reviewed by the EC. The VOR results often negatively impacted the proposal rankings, likely because the estimated return on investment was small or negative in many cases. Even though the scores changed following presentation of the VOR results, the information did not materially change the EC recommendation from an “approve” to “reject” decision (or vice versa) for any proposal in this study.

Although there was general acceptance of the VOR methodology and appreciation of its potential value for decision making, the study investigator team did find ongoing resistance to VOR from a minority of investigators, as well as concerns at the outset that required modification of the approach. An early complaint was that investigators were being unfairly “punished” in the comprehensive VOR results because of the very high cost of the drugs that were frequently being evaluated in the trials they were designing. These high‐cost drugs were often the primary factor causing negative VOR estimates, that is, the value of the expected clinical benefit from a trial was less than the expected costs needed to generate the benefits at commonly used thresholds of societal value (ie, $150 000 per QALY).^18^ The investigators’ argument was that understanding the clinical impact of the drugs superseded the economic impact, and that the cost of the drugs was out of their control and changing over time. In response, the research team created a “clinical VOR” result that excluded treatment costs. This issue would also suggest that our VOR educational materials should include more information about opportunity costs, especially in the context of high‐cost treatments.

The investigators also raised the concern that trials addressing treatments for uncommon cancers were unfairly disadvantaged compared to trials for more common cancers. In response, the investigator team presented VOR estimates for the average patient (in addition to population level) to allow comparisons independent of the size of the overall patient population. The intent of these changes was to create a VOR process that informed SWOG's decision‐makers and their VOR preference heterogeneity. Even with these modifications, a few EC members did not participate in the VOR training or evaluation components of the study. It is unclear whether this was due to lack of interest or actual resistance to the concept and approach. This resistance was reflected in the responses to two end‐of‐study survey study questions; 8% of respondents stated that the VOR materials hindered the evaluation process and 17% disagreed with a question asking about support for adding VOR to the proposal evaluation process. This reinforces the need for early engagement with the research organization and efficiency in the integration process to decrease undue burden.

Prior work by ourselves and other U.S. researchers evaluated VOR with healthcare stakeholders, but the evaluation took place outside of a specific decision‐making process.^7^, ^9^ Outside of the US, Claxton and Sculpher identified similar challenges to those that we encountered in a pilot study that applied VOR to inform policy decisions about research priorities in the United Kingdom.^4^ In general, the committees involved in reviewing the studies found that VOR results were “interesting and potentially useful,” although they did not have an impact on the decisions taken. Unfamiliarity with the methods was cited as an issue, as well as some questioning the quality and relevance of the models.^4^ Our findings are similar to past studies in that the barriers to adoption of VOR‐informed research prioritization are primarily cultural instead of technical.^19^

Our study has limitations which warrant consideration. The clinical experts within SWOG had difficulty providing estimates of the current uncertainty about the proposed treatment decision to be evaluated in the given protocol. To address this, we developed an expert elicitation survey and an alternative option based on historical data about how often SWOG trials met their study endpoints. To reduce the potential bias due to optimistic estimates in favor of the new interventions, the survey was provided to the entire disease committee, rather than just the proposal development team and included data about historical norms, that is, “Data from a review of cooperative group clinical trials from 1955 to 2006 indicate that these values are 60% and 25% on average, respectively.” Our study was also limited by the number of proposals that were evaluated by the SWOG EC during the prospective evaluation period. Our evaluation was limited to phase II and III studies that had comparator arms. Uncontrolled studies have value, but our minimal model VOR approach cannot easily accommodate these study designs. We were also limited to average executive committee scores due to the anonymous nature of the SWOG voting process, thus we were not able to evaluate individual‐level impacts. Finally, changes in the composition of the EC hindered our pre/post evaluations and thus limited our ability to assess changes in attitudes toward VOR over time.

There are number of potential areas for future research about the role and use of VOR in research prioritization. One such area would be to investigate the best methods for establishing estimates of the current uncertainty using expert elicitation or other methods. Another area is the potential impact of VOR later in the proposal evaluation process, that is, at the NCI level, where the final funding decision is made. Future researchers may wish to take the lessons gleaned from this study to determine whether VOR be feasible and acceptable in other cancer cooperative groups or other clinical trial settings in different disease areas.

In summary, we developed and implemented a VOR evaluation process for clinical trial proposals being developed for SWOG using collaborative engagement and an efficient minimal modeling approach. The process was feasible in a decision‐relevant time frame, impacted scores, and EC member opinions were mixed but mostly favorable. SWOG leaders currently consider a number of factors in research prioritization such as scientific validity, study feasibility, and potential impact on patients and patient care. We view VOR as complementary to these considerations, as well as providing a quantitative estimate that can help understand the impact of the study on cancer care decisions and outcomes. In line with previous work on this topic, engagement, education, and efficiency are essential to successful integration.

## CONFLICT OF INTEREST

None declared.
