# Supplementary material for: Integrating value of research into NCI Clinical Trials Cooperative Group research review and prioritization: A pilot study
Source: Cancer Med. 2018 Jul 20;7(9):4251–60. doi: 10.1002/cam4.1657 (PMC6144145; doi:10.1002/cam4.1657)
Supplement: Supplementary file 1 [file CAM4-7-4251-s001.docx]

SUPPLEMENTARY METHODS

*Determining the current level of uncertainty for VOR Model Parameters:*

We surveyed disease committee members to estimate the current level of uncertainty regarding the proposed trial (See Appendix S2: Expert elicitation survey). The survey had two key questions: 1) What is the probability the new treatment is equivalent or better than the control arm for and, 2) What is the probability the new treatment offers a substantial improvement over the control arm in (i.e. at least as well as the hypothesized effect size in the proposal)? These estimates were included if we had sufficient time to survey the committees and had 10 or more responses. In the absence of this data we used historical data on the outcomes of SWOG trials to inform the prior probability estimates. Data from a review of cooperative group clinical trials from 1955-2006 indicates that these values are 60% and 25% on average, respectively. [20-22] Using either of the survey or historical estimates, we derived a prior distribution of the treatment effect estimate for the proposed trial’s primary endpoint by fitting a distribution that matched the null and alternative hypothesis with the appropriate percentiles (e.g. 60^th^ and 25^th^ using historical data).

APPENDIX S1: Expert Elicitation Survey

# SWOG [ENTER DISEASE COMMITTEE] Survey (Study Chair: XXXX)

Greetings.

This activity is part of a PCORI funded project that is evaluating the use of value of information analyses to support research prioritization efforts within SWOG (<http://www.pcori.org/research-results/2013/structured-approach-prioritizing-cancer-research-using-stakeholders-and-value>).

The intent of this survey is to capture the current level of uncertainty regarding the proposed trial, [ENTER TRIAL CAPSULE NAME].  The responses will be used to calculate the expected value generated by conducting the trial as proposed.  The survey has two key questions: 1) What is the probability the new treatment is equivalent or better than the control arm for [ENTER TRIAL ENDPOINT]. (i.e. HR ≤ 1 in favor of new treatment); 2) What is the probability the new treatment offers a substantial improvement over the control arm in [ENTER TRIAL ENDPOINT] (at least as well as hypothesized in the capsule)?  The latter would indicate that the trial met its primary endpoint.  Data from a review of cooperative group clinical trials from 1955-2006 indicates that these values are 60% and 25% on average, respectively (Djulbegovic et al. 2008).

[OPTIONAL: ADD TEXT FROM CAPSULE]

## Question 1.

What is the probability the new treatment [ENTER TREATMENT NAME] is equivalent or better than the control arm[ENTER TREATMENT NAME] for [ENTER TRIAL ENDPOINT]. ([ENTER DATA FROM CAPSULE])  

## Question 2.

What is the probability the new treatment [ENTER TREATMENT NAME] offers a substantial improvement over the control arm [ENTER TREATMENT NAME] in [ENTER TRIAL ENDPOINT] (at least as well as hypothesized in the capsule) (i.e. ENTER DATA FROM CAPSULE)?

**INFORMATION FROM CAPSULE:**

[INSERT TEXT ON STUDY OBJECTIVES, JUSTIFICATION, AND STATISTICAL ANALYSIS PLAN]

APPENDIX S2: EC Baseline and End of Study Surveys

Assessment of Criteria for Ranking Study Capsules Executive Review Committee Baseline Survey

**Q1 – Q4** *Please provide the following information:*

**Q1.** *How long have you served on the Executive Review Committee?*

_______________years

**Q2.** *How long have you been a member of SWOG?*

_______________years

**Q3.** *What is your professional training?*

- MD
- PhD (please specify discipline, e.g. biostatistics, epidemiology, etc.) _____________________
- Other _______________

**Q4.** *What, if any, is your sub-specialty affiliation?*

- Hematologic malignancies
- Neuro-oncology
- Breast cancer
- Gynecologic cancers
- Thoracic cancers
- Gastrointestinal cancers
- Genitourinary cancers
- Head and Neck cancers
- Melanoma & other skin cancers
- Endocrine cancers
- Pediatric cancers
- Radiation Oncology
- Other ___________________

**Q5-Q15** *Below are a list of criteria that are typically used to review and prioritize study proposals. For each criterion, please rate the level of* ***Importance*** *that you assign to this factor (relative to the other factors) when assessing an individual study capsule and assigning a score. Please rate the criteria based on your experience as a member of the SWOG Executive Review Committee during the past 12 months (or your full tenure, if < 12 months).*

|  |  | **Not at all important (1)** | **Very low importance (2)** | **Slightly important (3)** | **Somewhat important (4)** | **Moderately important (5)** | **Very Important (6)** | **Extremely important (7)** |
| --- | --- | --- | --- | --- | --- | --- | --- | --- |
| **FEASIBILITY** | How well has the principle investigator addressed potential barriers to successful study implementation and completion? (e.g., potential patient accrual difficulties, competing studies, uncompensated study costs, etc.) |  |  |  |  |  |  |  |
| **CLINICAL IMPORTANCE** | Are the study results likely to have a significant clinical impact on some group of patients, regardless of the size of the affected population? |  |  |  |  |  |  |  |
| **SCIENTIFIC CONTRIBUTION** | Does the study test an important scientific concept or proof of principle that could be generalized to future studies? |  |  |  |  |  |  |  |
|  |  | **Not at all important (1)** | **Very low importance (2)** | **Slightly important (3)** | **Somewhat important (4)** | **Moderately important (5)** | **Very Important (6)** | **Extremely important (7)** |
| **RELATIVE COST/ RESOURCES** | What are the costs to conduct the study either at the level of the individual institution or at the level of SWOG? (this criterion does NOT refer to the cost of the disease or its treatment to the health care system) |  |  |  |  |  |  |  |
| **DISEASE BURDEN** | Is the condition or disease associated with a significant burden in the US population (in terms of prevalence, mortality, morbidity or individual suffering)? |  |  |  |  |  |  |  |
| **POTENTIAL FOR IMPROVING CLINICAL PRACTICE** | What is the likelihood that results from this study will lead to a change in clinical practice or clinical decision making, or confirms current practice remains best? |  |  |  |  |  |  |  |
| **TIMELINESS** | Will this study be able to be completed within a relatively short time-frame (i.e., < 5 years), so that changes in clinical practice will not render the findings obsolete? |  |  |  |  |  |  |  |
| **SUITABILITY OF THE STUDY FOR CONDUCT WTIHIN SWOG** | Is the study as designed well-suited for conduct within SWOG, or would it be better suited for conduct in another clinical study setting? |  |  |  |  |  |  |  |
|  |  | **Not at all important (1)** | **Very low importance (2)** | **Slightly important (3)** | **Somewhat important (4)** | **Moderately important (5)** | **Very Important (6)** | **Extremely important (7)** |
| **ROLE OF THE NCI** | Is there a current perception that the funding agency is particularly interested in this study topic? |  |  |  |  |  |  |  |
| **UNCERTAINTY REGARDING THE INTERVENTION EFFECT** | How much uncertainty exists regarding the effectiveness of the treatment/intervention prior to this planned study? |  |  |  |  |  |  |  |
| **ECONOMIC VALUE** | How do the expanded health benefits compare to the expected costs of the new intervention? Are the benefits likely to justify the increased cost? |  |  |  |  |  |  |  |

**Q 16.** *What is your prior level of experience with using Value of Information as a tool to aid in research priority setting?*

- No prior experience
- Some familiarity with the VOI literature
- Training only, no real-world application
- Limited experience (<1 year)
- Moderate experience (1-3 years)
- Extensive experience (>3 years)

**Q 17.**

|  | **Extremely dissatisfied (1)** | **Very dissatisfied (2)** | **Somewhat dissatisfied (3)** | **Neither satisfied nor dissatisfied (4)** | **Somewhat satisfied (5)** | **Very satisfied (6)** | **Extremely satisfied (7)** |
| --- | --- | --- | --- | --- | --- | --- | --- |
| How satisfied are you with the current decision-making process used to evaluate study capsules within the SWOG Executive Review Committee? |  |  |  |  |  |  |  |

PCORI Value of Information Study Executive Review Committee End of Study Survey

**Q1:**

How many years have you served on the Executive Review Committee?

**Q2**

How many years have you been a member of SWOG?

**Q3**

What is your professional training (select all that apply)?

|  | - MD |
| --- | --- |
|  | - PhD |
|  | - Other: |

**Q4**

What, if any, is your sub-specialty affiliation?

|  | - Hematologic malignancies |
| --- | --- |
|  | - Neuro-oncology |
|  | - Breast cancer |
|  | - Gynecologic cancers |
|  | - Thoracic cancers |
|  | - Gastrointestinal cancers |
|  | - Genitourinary cancers |
|  | - Head and neck cancers |
|  | - Melanoma and other skin cancers |
|  | - Endocrine cancers |
|  | - Pediatric cancers |
|  | - Radiation oncology |
|  | - Other: |

**Q5**

How many VOI analyses have you reviewed as part of the Executive Review committee?

**Q6**

How would you rate your knowledge of value of information methods?

|  | - No knowledge |
| --- | --- |
|  | - Limited knowledge |
|  | - Moderate knowledge |
|  | - Good knowledge |
|  | - Excellent knowledge |

**Q7**

How would you rate your confidence in interpreting VOI evidence

|  | - No confidence |
| --- | --- |
|  | - Low |
|  | - Moderate |
|  | - High |
|  | - Very High |

**Q8-Q15**

Below is a list of questions related to the value of information capsule evaluation process that was recently studied within SWOG and the EC. For each statement, please rate the level of **agreement** that you assign to this statement

|  | Disagree (1) | Somewhat disagree (2) | Neither agree nor disagree (3) | Somewhat agree (4) | Agree (5) | Not applicable (6) |
| --- | --- | --- | --- | --- | --- | --- |
| The VOI research team addressed my input before incorporating VOI into the capsule process |  |  |  |  |  |  |
| I received sufficient VOI training to understand the purpose of VOI and its relevance to SWOG studies |  |  |  |  |  |  |
| The VOI material provided to the EC triage meetings was easy to understand |  |  |  |  |  |  |
| The material provided during the EC triage meetings was appropriate in length |  |  |  |  |  |  |
| Overall, the VOI capsule evaluation process was useful for my decision making |  |  |  |  |  |  |
| The VOI evaluation materials helped the SWOG capsule evaluation process |  |  |  |  |  |  |
| The VOI evaluation materials hindered the SWOG capsule evaluation process |  |  |  |  |  |  |
| I support adding VOI into the SWOG's capsule evaluation process after the evaluation period. |  |  |  |  |  |  |

**Q16**

|  | Extremely dissatisfied (1) | Very dissatisfied (2) | Somewhat dissatisfied (3) | Neither satisfied nor dissatisfied (4) | Somewhat satisfied (5) | Very satisfied (6) | Extremely satisfied (7) |
| --- | --- | --- | --- | --- | --- | --- | --- |
| How satisfied are you with the VOI-informed decision-making process used to evaluate study capsules within the SWOG Executive Review Committee? |  |  |  |  |  |  |  |

**Q17-Q28**

Below are a list of criteria that are typically used to review and prioritize study proposals. For each criterion, please rate the **level of importance** you assign to this factor (relative to other factors) when assessing an individual study capsule and assigning a score.

|  | Not at all important (1) | Very low importance (2) | Slightly important (3) | Somewhat important (4) | Moderately important (5) | Very important (6) | Extremely important (7) |
| --- | --- | --- | --- | --- | --- | --- | --- |
| Feasibility: How well has the principle investigator addressed potential barriers to successful study implementation and completion? (e.g., potential patient accrual difficulties, competing studies, uncompensated study costs, etc.) |  |  |  |  |  |  |  |
| Clinical importance: Are the study results likely to have a significant impact on some group of patients, regardless of the size of the affected population? |  |  |  |  |  |  |  |
| Scientific contribution: Does the study test an important scientific concept or proof of principle that could be generalized to future studies? |  |  |  |  |  |  |  |
| Relative cost/resources: What are the costs to conduct the study at the level of the individual institution or at the level of SWOG? (this criterion does NOT refer to the cost of the disease or its treatment to the health care system) |  |  |  |  |  |  |  |
| Economic value: How do the expanded health benefits compare to the expected costs of the new intervention? Are the benefits likely to justify the increased cost? |  |  |  |  |  |  |  |
| Disease burden: Is the condition or disease associated with a significant burden in the US population (in terms of prevalence, mortality, morbidity or individual suffering)? |  |  |  |  |  |  |  |
| Uncertainty regarding the intervention effect: How much uncertainty exists regarding the effectiveness of the treatment/intervention prior to this planned study? |  |  |  |  |  |  |  |
| Potential for improving clinical practice: What is the likelihood that results from this study will lead to a change in clinical practice or clinical decision making, or confirm current practice remains best? |  |  |  |  |  |  |  |
| Timeliness: Will this study be able to be completed within a relatively short time-frame (i.e., <5 years), so that changes in clinical practice will not render the findings obsolete? |  |  |  |  |  |  |  |
| Suitability of the study for conduct within SWOG: Is the study as designed well-suited for conduct within SWOG, or would it be better suited for conduct in another clinical study setting? |  |  |  |  |  |  |  |
| Role of the NCI: Is there a current perception that the funding agency is particularly interested in this study topic? |  |  |  |  |  |  |  |
| Value of information: What is the expected clinical and economic value of conducting the study as proposed? |  |  |  |  |  |  |  |

**Q29**

Please take a moment to let us know any comments or suggestions you might have regarding the VOI capsule evaluation process.

**Value of Information - Educational Brief**

**Preface**

We are developing an approach using an emerging methodology, ‘**Value Of Information’ (VOI)** analysis, to estimate the societal return on investment of proposed clinical trials. We are enlisting the help of SWOG members to assess the usefulness of this approach. **Our goal is to develop a tool that will be accepted and useful to SWOG members.**

The purposes of this brief are to provide a summary of VOI analysis and an overview of how we’d like to work with you going forward. We hosted an in-person training session at the 2014 Spring SWOG meeting that present these methods in further detail and provided examples using historical trial proposals reviewed by the Executive Committee. A recorded copy of this training session can be accessed online [URL TO BE INSERTED HERE]. We welcome your feedback on these materials.

**Why estimate the ‘Value of Information’ for trial proposals?**

Faced with a promising new cancer treatment but limited evidence, should we adopt it, reject it, or conduct a new trial? SWOG and NCI have finite resources to invest in clinical trials and are often faced with making difficult decisions regarding which trials to support. For example, should SWOG commit to a large, long-term trial or invest in multiple smaller trials? Would a longer trial with a “hard” endpoint like survival provide better evidence to support decision-making versus a shorter trial using a surrogate endpoint? The Institute of Medicine (IOM) report, *A National Clinical Trials System for the 21^st^ Century: Reinvigorating the NCI Cooperative Group Program*, called for formal methods to prioritize clinical trials in cancer to ensure that limited public funds are used in ways that are likely to have the greatest impact on patient care. Developing a quantitative yet transparent and stakeholder-driven process may enable SWOG to achieve greater success in trial funding and benefits to patients. With proper adaptation and implementation, we believe that estimating the Value of Information for trial proposals can help SWOG decision makers target their resources to clinical problems that would yield the highest clinical and economic return to patients and society for the investment.

**What is ‘Value of Information’ analysis?**

Value of Information is a measure of the value of reducing treatment decision uncertainty. When we conduct clinical trials, the information provided on relative effectiveness and safety improves the chances we will make optimal treatment decisions in the future. For example, a small phase 2 trial has a wide confidence interval around the treatment effect. If we were to make treatment decisions based on this small trial, the probability that we would be making the optimal decision would be fairly low. By conducting a larger trial, our chances of making correct treatment decisions is increased – regardless of whether the trial is ‘positive’ or ‘negative.’ Value of information analysis captures this value by estimating the likely future improvements in patient outcomes and healthcare resources.

VOI analyses can be used to estimate the amount a decision maker would be willing to pay for perfect information^[[1]](#footnote-1)^. This amount is the most a decision maker would pay for a trial that would eliminate the chance of making a wrong decision. This hypothetical construct represents an upper bound value for the particular research question (it requires a trial with an infinite sample size). VOI analyses can also be used to estimate a more realistic situation: the value of information from a future clinical trial with a finite sample size^[[2]](#footnote-2)^. A clinical trial with a finite sample size will not provide perfect information about a treatment, but it reduces uncertainty and therefore improves decision-making and benefits patients. We plan to estimate both values (upper bound value and value with given sample size) for SWOG’s clinical trial proposals.

In the context of VOI, the economic value of a clinical trial, is a function of four key elements: 1) the current level of decision uncertainty (i.e. the probability that we are making suboptimal decisions based on current knowledge), 2) how much new information will be collected in the trial, 3) the consequences of making a suboptimal decision in terms of a patient’s life expectancy, quality of life, or healthcare costs, and 4) the number of future patients likely to face the decision. Consequently, the VOI will be high when there is a lot of uncertainty about the decision, the clinical and/or economic consequences of making a suboptimal choice are large, and the affected population is large.

**Example: RxPonder clinical trial S1007**

Preliminary evidence suggests that using the Oncotype Dx Breast Cancer Assay to guide chemotherapy treatment in node-positive, HR-positive breast cancer potentially could spare thousands of women from chemotherapy-related morbidity and dramatically reduce expenditures. But this evidence is uncertain: given limited data it is very possible that using the assay could produce worse outcomes, higher costs, or both. The results from SWOG’s RxPonder trial (S1007) are expected to provide substantial value to society because current evidence is very uncertain, the clinical and economic consequences of a suboptimal decision are large, and a considerable number of patients are expected to face the decision to use, or not, the Oncotype Dx assay. In a previous analysis, we estimated the societal value of the RxPonder trial to be between $450 million to $1.05 billion^[[3]](#footnote-3)^.

Does the RxPonder trial represent a good value? The RxPonder trial is expected to cost the NIH approximately $27 million, which means the trial will provide a 17 to 39-fold return on investment. Such a high return suggests that the RxPonder trial is a very good research investment; however, we do not know whether funding a different trial or set of trials could have provided an even higher return. This is a question we plan to explore in our proposed study.

**How will we estimate the VOI for a trial proposal?**

VOI analysis involves the application of methods from economic theory and decision analysis to estimate the clinical and economic value of performing additional research to better understand the safety, efficacy, and cost of technologies and medical interventions. These analyses are performed by developing a mathematical model of the treatment options and their projected clinical outcomes. Estimates of clinical probabilities, quality of life, and healthcare costs are derived from the literature and expert input. We then conduct simulations to estimate the impact that a new trial will have on the probability of choosing the optimal treatment and the resultant clinical and economic consequences.

We will use:

- Simulation models to project the long-term clinical and economic outcomes of patients in the trial. These models formally capture our current knowledge and level of uncertainty about an intervention’s clinical and economic impact by extrapolating from a trial’s primary endpoint(s) to comprehensive outcome measures, such as Quality Adjusted Life Years (QALYs) and lifetime costs. We must make a number of important assumptions for these extrapolations and will be seeking your input on how best to do so.
- Population-level projections to calculate the expected number of current and future individuals who will face a choice about which treatment to use and will therefore benefit from the additional information.

**Proposed Approach**

We plan to use VOI analysis to estimate the value for late-phase clinical trial proposals reviewed by the Breast, Genitourinary, and Gastrointestinal disease committees. These committees were chosen given their high volume of capsules and willingness to participate. We hypothesize that providing Executive Committee members with estimates of the societal value at the time the trial is reviewed will help them select the most promising trials and therefore maximize the expected return of the entire research portfolio – as well improve the funding success of SWOG trials. Over the next 2-3 years, we will formally evaluate whether providing VOI results to Executive Committee members as they review trial proposals influences their scoring and ranking of the proposals, their satisfaction with the decision-making process, and ultimately the rate of NCI approval for SWOG’s trials.

**What role will SWOG members play in developing and evaluating this methodology?**

The primary role of SWOG investigators will be to provide us with input on the estimates for treatment safety and effectiveness. Specifically, we need to know the long-term consequences of different treatments, such as the impact on progression-free survival, the association between progression-free survival and overall survival, or the impact of adverse events on a patient’s quality of life. We know these issues are considered carefully when designing and reviewing trial proposals, and **we are specifically interested in your thoughts and feedback regarding how best to address these issues efficiently in building our models.**

**What are the next steps?**

At the Fall 2014 SWOG Biennial meeting in Chicago, we will present the results of approximately 12 previously reviewed trial capsules from the Breast, Genitourinary, and Gastrointestinal committees. During this meeting we will actively solicit your questions, thoughts, and concerns regarding the application of these methods. Shortly after the meeting, we will ask you to complete a brief online survey to gauge your confidence in and satisfaction with the proposed methods and any modifications made in response to concerns raised in the meeting and beforehand.

**Where I can learn more?**

Examples of value of information analyses in oncology:

- Wong WB, Ramsey SD, Barlow WE, Garrison LP, Jr., Veenstra DL. The value of comparative effectiveness research: projected return on investment of the RxPONDER trial (SWOG S1007). Contemp Clin Trials. 2012 Nov;33(6):1117-23.
- Carlson JJ, Thariani R, Roth J, Gralow J, Henry NL, Esmail L, et al. Value-of-information analysis within a stakeholder-driven research prioritization process in a US setting: an application in cancer genomics. Medical decision making : an international journal of the Society for Medical Decision Making. 2013 May;33(4):463-71.
- Havrilesky LJ, Chino JP, Myers ER. How much is another randomized trial of lymph node dissection in endometrial cancer worth? A value of information analysis. Gynecologic oncology. 2013 Oct;131(1):140-6.

Value of information theory and research prioritization:

- Implementing Comparative Effectiveness Research: Priorities, Methods, and Impact. *Brookings Institute.* June 2009. Accessible from: <http://www.brookings.edu/~/media/research/files/papers/2009/6/09%20cer%20mclellan/0609_health_care_cer.pdf>
- A Pilot Study of Value of Information Analysis to Support Research Recommendations for NICE. University of York. June 2005. Accessible from: <http://www.york.ac.uk/media/che/documents/papers/researchpapers/rp4_Pilot_study_of_value_of_information_analysis.pdf>

(1) ([3](#_ENREF_3))

1. This is known as the Expected Value of Perfect Information, or EVPI [↑](#footnote-ref-1)
2. This is known as the Expected Value of Sample Information, or EVSI [↑](#footnote-ref-2)
3. Wong WB, Ramsey SD, Barlow WE, Garrison LP, Jr., Veenstra DL. The value of comparative effectiveness research: projected return on investment of the RxPONDER trial (SWOG S1007). Contemp Clin Trials. 2012 Nov;33(6):1117-23. [↑](#footnote-ref-3)
